# Sustainable Wearable Sensors for Plant Monitoring and Precision Agriculture

**DOI:** 10.1021/acs.analchem.5c01565

**Published:** 2025-06-20

**Authors:** Samiris Côcco Teixeira, Nathalia O. Gomes, Taíla Veloso de Oliveira, Nilda F. F. Soares, Paulo A. Raymundo-Pereira

**Affiliations:** † Food Technology Departament, 28120 Universidade Federal de Viçosa, Avenida PH Holfs s/n, Campus Universitário, 36570-000 Viçosa, Minas Gerais, Brazil; ‡ Sao Carlos Institute of Chemistry, University of Sao Paulo, CEP 13566-590 Sao Carlos, SP, Brazil; § Nanotechnology National Laboratory for Agribusiness (LNNA), Embrapa Instrumentation, CEP 13561-206 São Carlos, SP, Brazil; ∥ Sao Carlos Institute of Physics, University of Sao Paulo, CEP 13560-970 Sao Carlos, SP, Brazil

## Abstract

Wearable sensors are emerging and innovative tools in the realm of agriculture, offering new opportunities for sustainable plant monitoring practices. This perspective explores wearable sensor technology in plant monitoring to promote environmental sustainability and enhance agricultural productivity. Wearable sensors, capable of continuously tracking plant health indicators such as salinity, diseases, metabolites, pH, ions, pathogens, pesticides, parasites, phytohormones, nutrient status, moisture levels, and pest activity, provide real-time information to make precise and timely decisions. Farmers can use the diverse collected data to enhance resource use, reducing waste and the environmental impact of agricultural practices. Here, we highlight the current advancements in wearable sensor technology and explore potential applications in diverse agricultural settings, with the challenges and opportunities to be addressed to fully implement by the farming community. We also emphasize the sustainable and biodegradable substrates/supports relying on eco-friendly polymeric materials for the fabrication of cost-effective, flexible, durable, stable, and easily deployable sensor systems, which can be extensively applied by the agrifood sector. We provide a forward-looking perspective on how wearable sensors can contribute to more sustainable and efficient plant monitoring practices in precision agriculture. Given the disruptive innovation, wearable plant sensors were highlighted as Top 10 Emerging Technologies by World Economic Forum in 2023.

## Graphical abstract

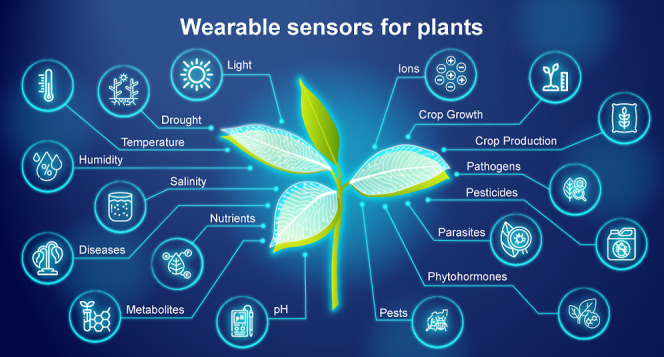

## Introduction

The global demand for food is rapidly increasing and perhaps will continue for decades, propelled by expanding global population, estimated to be 9.8 billion by 2050 and 11.2 billion by 2100.^1–3^ The crop yields should increase 100–110% between 2005 and 2050 to avoid a scenario of food insecurity.^4^ Farmers are facing diverse challenges, such as crop vulnerability, extreme temperatures, soil degradation, and drought—issues that are expected to worsen with global warming.^4^ Safeguarding plant health is crucial for adapting to climate change, managing water resources effectively, and improving crop yields.^4^ The loss of crops exceeds ∼220 billion dollars,^5^ and the Food and Agriculture Organization (FAO) estimates that 40% of global crop productivity is lost each year due to emerging plant diseases and environmental stressors related to climate changes impacting agriculture, compromising nutrition, and threatening food security worldwide.^6^ Pests, pathogens, and extreme weather events, including floods, droughts, and heat stress, pose serious threats to plant health and the agricultural ecosystem, endangering both productivity and sustainability.^2^

With the aim of achieving a better life and future for all, the pivotal role of the agrifood sector in increasing productivity at the plant level sustainably has become the main force to drive the world into a new era, which was established by the 2030 Agenda to reduce crop losses and poverty.^6^ Enhanced sustainable agricultural practices are essential to ensure high yields that employ minimal inputs and are nondestructive to the land.^7^ With recent advances in sensing technology, plant health monitoring is an emerging field with great potential to create more productive systems of agriculture to increase yields and decrease environmental impact.^7^ Conventional plant health monitoring and environmental factors employ remote and contactless sensing technologies including proximal optical sensors, spectroscopy, machine vision systems, imaging techniques, and drones.^2,3^ However, conventional monitoring technologies have limitations, including low spatial and temporal resolution, discontinuous measurements, poor sensitivity, stability, and reliability, rendering them less effective for the accurate and continuous monitoring of plant growth, microclimate, and plant organ development.^2,3^

In contrast to traditional precision agriculture methods, sensory technology and innovative data collection tools, harnessed to monitor and regulate crucial parameters conducting plant health, quality, stress responses, and morphological, biochemical, and physiological traits in a dynamic environment,^2,3^ have emerged to equip farmers with informed decision-making capabilities.^8^ Wearable sensors have been developed to noninvasively and continuously monitor human health in biomedical, nutritional, and fitness applications.^9–12^ However, the cutting-edge wearable sensing application in agriculture is still in its initial stage.^12^ The employ of wearable sensor technology for continuous sensing and remote probing of the plant vitality may meet the requirements toward routine practice in a nondestructive way.^13^

Plant-wearable sensors or wearable plant sensors, i.e., wearable sensors for plant monitoring, are small, stretchable, and miniaturized analytical devices capable to be directly attached on several plant parts, like stems, food skin, and leaves, for continuous monitoring of temperature and humidity,^14^ dehydration,^13^ biomarkers,^15^ diseases,^16^ nutrient levels,^17^ pesticides,^18,19^ and diverse biotic and abiotic stress,^8,20^ as illustrated in Figure 1. Wearable sensor technology for plants offers a cutting-edge tool for on-site, noninvasive, nondestructive, fast, and decentralized analysis toward precision agriculture and food safety applications, providing real-time parameters into plant health and environmental conditions.^18,19,21^ The collected data have great potential to optimize yields, reduce waste, detect early signs of disease and pests, and minimize the environmental impact of agriculture, as they can noninvasively, nondestructively, and continuously monitor the physiological and health status of plants.^8,22^ Although several challenges remain, wearable sensors for plants will revolutionize the agri-food sector enhancing crop production, management, and food quality, enabling farmers to make fast decisions regarding fertilizer application, irrigation schedules, and environmental adjustments.^2,23^ The wearable sensor capability to remotely monitor enhanced convenience and efficiency in plant healthcare certainly will revolutionize plant cultivation and maintenance with automatization of the agriculture industry implementing robots and autonomous systems integrated to the advanced technologies, including Big Data, machine learning (ML), artificial intelligence (AI), cloud computing, and the Internet of Things (IoT).^24–26^ Given the high expectation of enhance plant health and improve agricultural productivity revolutionizing the agrifood sector, wearable plant sensors or plant-wearable sensors were included in the Top 10 Emerging Technologies by World Economic Forum in 2023.

**Figure 1:**
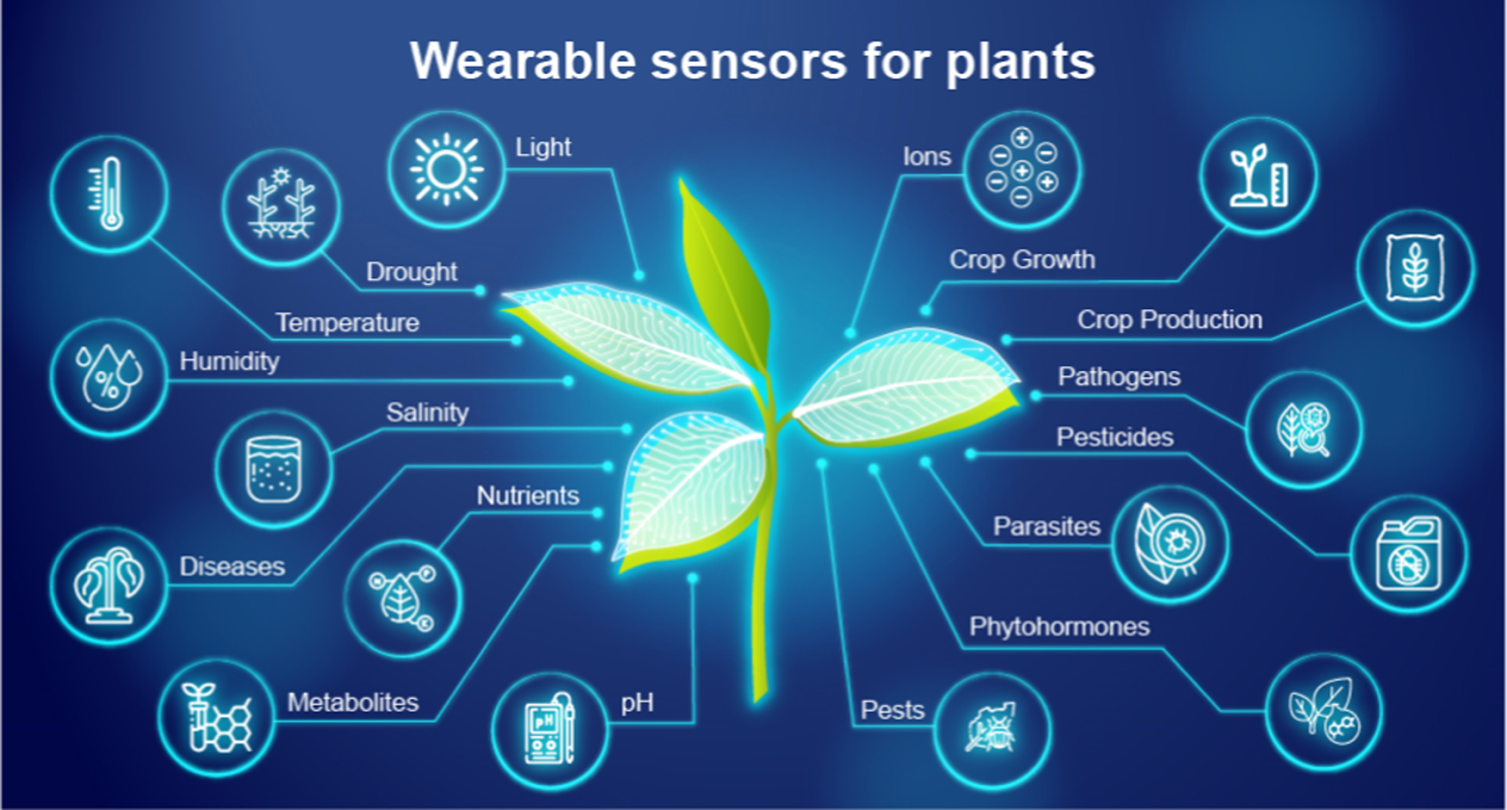
Schematic illustration of applications with sustainable wearable sensors for plant monitoring and precision agriculture.

In contrast to rigid elements, which can damage vulnerable parts and affect normal physiological processes of the plants,^2^ a wearable sensor requires flexible, conformable, and portable properties, allowing direct contact with irregular and curvilinear surfaces on the skin of stems,^4^ leaves,^19^ fruits, and vegetables surface^18^ without any harm at the plant/sensor interface.^3^ The wearable analytical tool offers compatibility for diverse structures of plants, i.e., stems,^4^ leaves,^19^ and the surfaces of fruits and vegetables,^18^ and also strongly adheres to the structure surfaces to achieve continuous long-term monitoring of environmental and plant health status parameters.^3^ However, most sensing devices are produced on substrates/support of non-degradable traditional petrochemical-based materials, e.g., poly­(ethylene terephthalate) (PET),^27^ nitrile gloves,^21,28^ polydimethylsiloxane (PDMS),^29^ polyester (PE),^27^ poly­(ether sulfone) (PES), poly­(ethylene naphthalate) (PEN), poly­(ether ether ketone) (PEEK), poly­(ether imide) (PEI), styrene–ethylene/butylene–styrene, or poly­(imides) (PI),^30^ and biomass-based non-biodegradable materials, e.g., polyethylene (Bio PE), polypropylene (Bio PP), polyurethane (Bio PU), polyamide (Bio PA), Bio PET, and polyethylene furanoate (Bio PEF),^31^ which are unsustainable materials and need a long time for degradation.^32^ Economic, environmental, and safety issues due to the massive use of flexible devices produced on non-degradable plastic polymers of chemical products derived from petroleum and biomass have intensified research on materials obtained from bioresources to find a promising eco-friendly, biodegradable, and sustainable alternative.^33^

Contrary to the non-degradable plastics, sustainable, biocompatible, and biodegradable materials hold a smaller market share, despite their potential to reduce the environmental effects of plastic production. The market for biodegradable sensors illustrated in Figure 2 projects an expressive increase due to the growing demand for eco-friendly monitoring solutions and sustainable agricultural methods worldwide. Data extracted from Next Move Strategy at https://www.nextmsc.com/report/biodegradable-sensors-market accessed in July, 2024. Figure 2 also highlights the regions of North America, Europe, Asia-Pacific, and Latin America with growing biodegradable sensor markets due to increasing interest for cutting-edge technology and robust agricultural industries, while North America is driven by environmental concerns and sustainable government programs. Technologies capable of decreasing the effects on the environment while increasing productivity and efficiency in agricultural and ecological management are boosted by government policies owing to the need to replace non-degradable traditional sensors with biodegradable devices.

**Figure 2:**
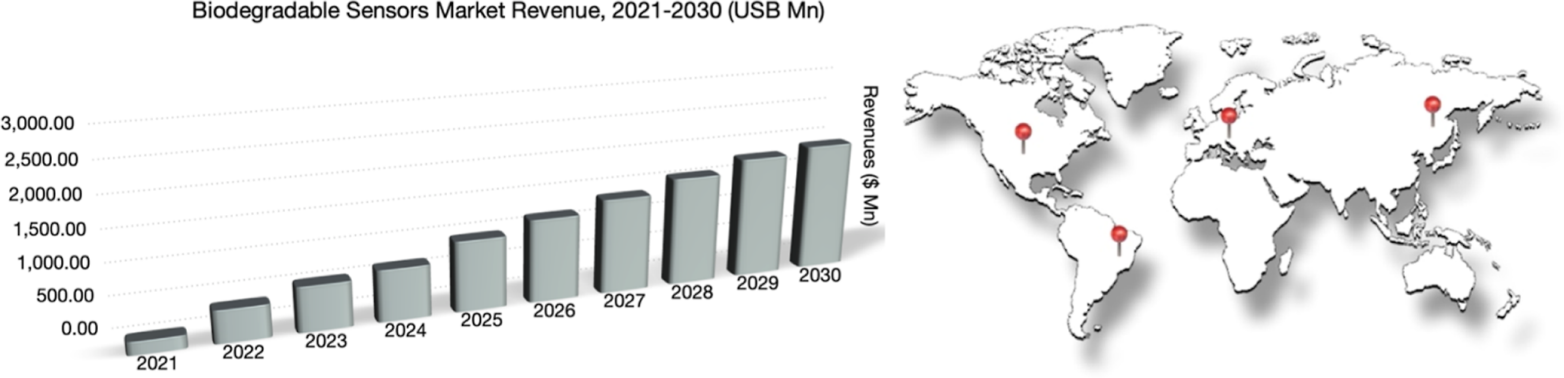
Prospect of global biodegradable sensor markets.

Biodegradable and biocompatible wearable sensors for plant monitoring are a pivotal improvement in sustainable agriculture and an environmentally friendly alternative to the imperative demand to replace non-degradable gadget,^34,35^ offering a solution by utilizing bio-based materials from renewable resources, e.g., polylactic acid (PLA),^18,33^ starch (SC)^36^ and cellulose derivatives.^19,32,37,38^ Most published studies in the wearable sensors area focus on using biodegradable materials as modifiers, components of composites, sensing elements, or matrices. In contrast, our perspective focuses on the use of flexible and sustainable substrates/supports as green alternatives to non-degradable-based plastics, a prominence topic less explored but which is gaining attention due to its outstanding significant economic, social, and environmental relevance.^32^ Additionally, they also lack a comparison with devices made from non-biodegradable substrates in terms of analytical performance, possibly because non-biodegradable materials tend to offer superior sensitivity, usability, and cost.^32^ The aim of this perspective is to enhance our understanding of the challenges involved in the development and adoption of biodegradable plant-wearable sensors for sustainable sensing technologies. By highlighting the main challenges, we strive to make significant contributions to the current conversation on sustainable sensing technologies in a society increasingly dependent on plastic substitutes.

## Components of Sustainable Plant-Wearable Sensors

The sustainable devices are a rapidly developing field interfacing materials science, electronics, chemistry, biology, and agriculture. With the goal to transform the agrifood sector, wearable sensors provide a long-term, environmentally friendly way to track continuously plant health conditions while reducing device impact after use.^3^ Plant-wearable sensors contain two main elements, i.e., a sensing element and a transparent flexible or stretchable support/substrate to avoid interference on natural growth and development by attaching devices directly on plant organs.^8^ The selection of the sensing layer and detection technique directly impacts the analytical performance.

Rapid and direct fabrication techniques for plant-wearable sensors include physical and chemical methods, e.g., 3D printing, inkjet printing, coating, direct writing (photolithography and plasma etching), deposition, blending, spinning, electroplating, and serigraphy (screen-printing),^8,39^ in which the devices are directly manufactured on a support or substrate with controlled morphologies, programmable compositions, and designable patterning, consequently reducing energy and time consumption and waste generation during preparation.^8,40^ The conductive material converts the sensing material’s response into a readable signal (transduction unit), which can include current (*I*), voltage (*V*), impedance (*Z*), resistance (*R*), capacitance (*C*), charge (*Q*), or electrical potential (*E*).^8^

The choice of the sensing layer depends on the specific target, analytical technique, sample, and detection requirements of tracking plant health. The use of nanostructures plays a decisive role to achieve high sensitivity, stability, robustness, a low detection limit, prolonged lifetime storage, and a wide range of linearity with interference-free.^41,42^ The sensors functionalization has been conducted utilizing diverse strategies, including sensing metallic materials (e.g., gold, titanium, platinum, silver, and copper),^43^ carbon nanomaterials (e.g., carbon black (CB),^44^ Ag@C nanocables (NC),^41^ carbon spherical shells (CSS),^42^ carbon conducting ink,^19^ Printex carbon nanoballs (PCNB)^45^), Prussian Blue nanoparticle (PBNP),^46^ and gold nanoparticles (AuNPs).^47^ Carbon-based nanomaterials have been highlighted for sensor applications due to their exceptional mechanical and electrical features.

The sensors are made to identify a broad range of compounds that are essential to the health and development of plants, as illustrated in Figure 1. These could include pollutants, fertilizers, insecticides, gases like carbon dioxide and oxygen, disease, and more.^13^ Wearable plant sensors work well with the latest developments in sensor technology to provide noninvasive, real-time monitoring capabilities,^19^ allowing prompt interventions to maximize agricultural productivity and environmental sustainability.^3,48^

The entire sensing arrangement perhaps could be complemented with a transmission system and advanced data processing. The sensor with a wireless or wired data communication function may be designed and assembled to realize the communication between wearable sensing systems and smartphones, tablets, or laptops.^8^

Plant-wearable sensors can be connected, allowing real-time data transmission to a centralized cloud-based platform.^20^ To monitor crop health, growth rates, and yield potential, the collected data are analyzed using ML, IoT, and other advanced data analytics techniques.^20^ Producers can use the collected information to make decisions on crop management.^20^ Figure 3 illustrates a full wearable sensing system with six components: substrate, conductive layer, functionalization, application, transduced signal transmission, and advanced technologies for data processing. Understanding the fundamental concepts and principles of work is crucial to guide the creation of sustainable wearable sensors with enhanced analytical performance with high sensitivity and selectivity, robustness, fast response time, satisfactory storage time, and improved stability for diverse analytes.

**Figure 3:**
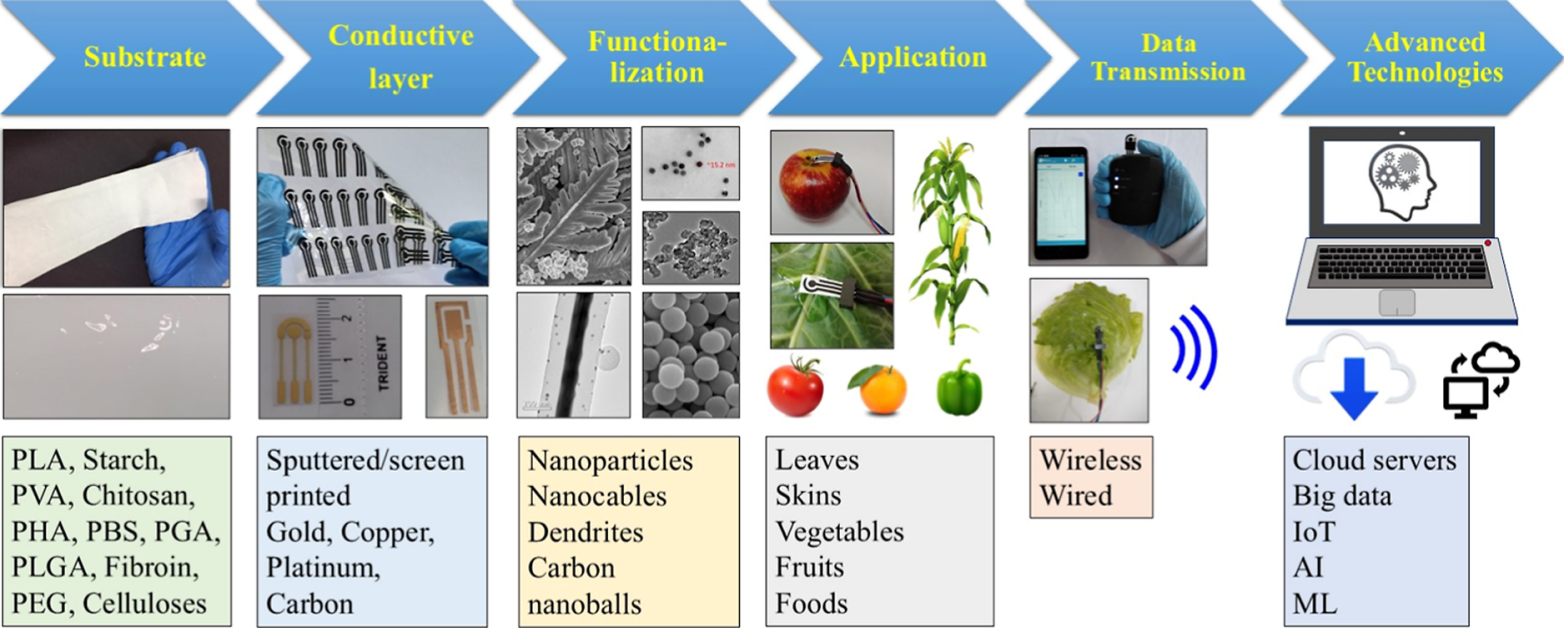
Schematic representing a fully integrated sustainable wearable sensor system to monitor plants.

## Biodegradable and Sustainable Substrates for Plant-Wearable Sensors

The substrate in which sensing elements are embedded is an electrically inert support essential to create sustainable wearable devices. The substrate layer, due to its larger thickness and area compared to other component layers, contributes significantly to the device’s overall weight and electronic waste.^49^ Substrate materials play a crucial role on device degradation and stability.^49^ Selecting suitable substrates for constructing high-performance biodegradable devices with controlled operational lifespans requires careful evaluation of parameters such as swelling rate, dissolution rate, and mechanical robustness.^49^

Bio-based polymeric materials such as cellulose acetate (CA), methylcellulose (MC), hydroxypropyl methylcellulose (HPMC), carboxymethylcellulose (CMC), PLA, chitosan (CN), SC, and poly­(vinyl alcohol) (PVA) have garnered significant attention as substrate/support for biodegradable devices due to their exceptional biocompatibility, environmental sustainability, abundance, and flexibility.^32,50^ With special highlight, cellulose, the most abundant natural polysaccharide on Earth, and its derivatives have emerged as a promising substrate for biodegradable sensors due to their favorable degradation behavior in physiological environments, high thermal stability, excellent biocompatibility, flexibility, and transparency.^19,37,38^ However, naturally derived substrates present challenges, including variability in mechanical properties and degradation rates.^49^ Polymers fabricated under controlled conditions can offer predictable and consistent mechanical and disintegration features.^49^ The potential limitations can be mitigated by utilizing biodegradable synthetic polymers, which the most commonly employed in sustainable sensors include PLA, PLGA, PLLA, PCL, PGS, and PVA.^49^ Figure 4 illustrates the chemical structures of several synthetic and natural biodegradable polymers.^49,51^

**Figure 4:**
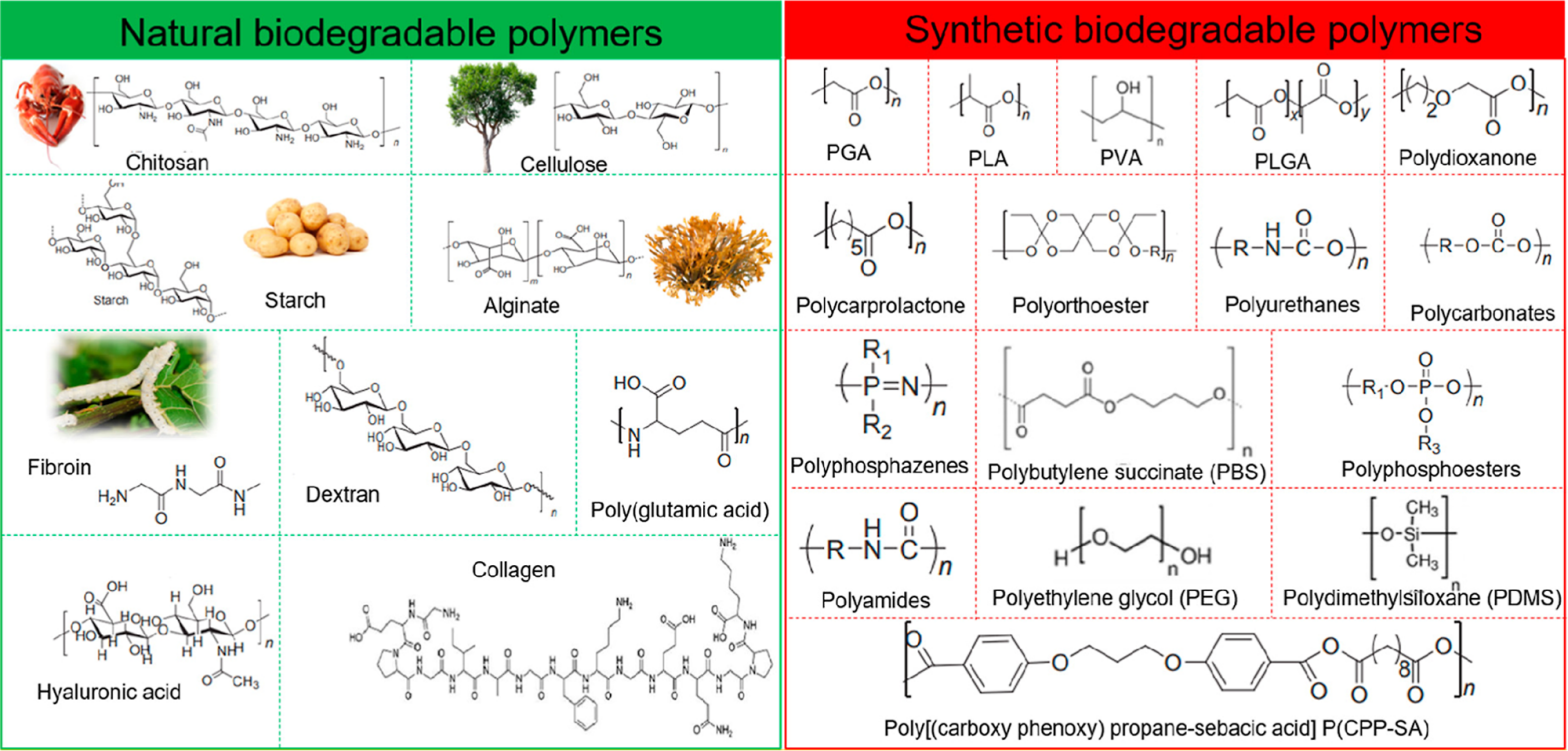
Chemical structures of diverse natural and synthetic biodegradable polymers. Reproduced from Hosseini, E. S.; Dervin, S.; Ganguly, P.; Dahiya, R. Biodegradable Materials for Sustainable Health Monitoring Devices. *ACS Appl. Bio Mater*. 2021, 4(1), 163–194, (ref 51). Copyright 2021 American Chemical Society.

One recent advancement toward environmentally friendly plant monitoring methods has been the invention of biodegradable sensors to collect crucial parameters for plant health and growth providing a novel way to address concerns on waste accumulation and environmental impact.^32,49,52,53^ The wearable sensors produced on biodegradable polymer-based substrates can be engineered to track factors, as illustrated in Figure 1. This section will examine biodegradable polymeric materials to produce substrates/supports for wearable sensors for plant monitoring applications. Diverse types of sustainable and biodegradable polymeric materials are discussed below. PLA and CA have been used to sustainable and biodegradable wearable sensors for plant monitoring, precision agriculture, and food security applications.^18,19^ Poly­(hydroxy alkanoate)­s (PHAs), polybutylene succinate (PBS), and poly­(glycolic acid) (PGA) are used for food/agriculture applications such as micronutrient release and food packaging applications; however, they still were not used to wearable sensors, paving the way to new opportunities to create analytical devices with unique features in wearable sensor applications.

## Polylactic Acid

PLA is a sustainable and biodegradable polymer made from renewable resources, including sugarcane, SC, and other plant-based sources. PLA exhibits remarkable properties such as mechanical strength, elasticity, biocompatibility, stiffness, and processability, with applications ranging from biomedical devices to packaging.^54–56^ The biodegradability of PLA under a suitable environment increases its attractiveness as an excellent eco-friendly candidate to be used as support/substrate in several applications, including plant health monitoring, due to the adaptability to individual needs and environmental friendliness.

To address the issues of downsizing, biodegradability, mobility, and dependability that arise in the creation of precision agriculture sensor networks, Gopalakrishnan et al. (2022)^57^ carried out an inventive study introducing the degradable intelligent radio transmitting sensor (DIRTS), a cutting-edge device made to satisfy these specifications employing a biodegradable substrate. DIRTS was created to accomplish biodegradability and miniaturization by using additive manufacturing processes and electrically tiny antenna (ESA) technology. To find the best size for sensors working within the ideal frequency range for soil monitoring throughout changing moisture conditions, comprehensive research on ESAs was carried out. Biodegradable and radio frequency-compatible materials were found for sensor design and production. The authors used scalable additive manufacturing techniques, laser processing adhesive-backed substrates, and 3D printing biodegradable substrates. For real-time measurements, a lightweight, portable readout system was built and fitted with a drone to evaluate the sensor operation in both laboratory and field conditions. Drone-based measurements were carried out in agricultural fields to showcase the integration possibilities with agricultural drone technology and to demonstrate the practical usefulness of DIRTS. The authors concluded by outlining a methodical technique for determining sensor degradation rates in soil, enabling field-based calculation of sensor lifetime and decomposition time. This study demonstrated the effective creation of DIRTS using PLA, offering a viable methodology to track plant health and enabling additional study in the field.

Gomes et al. (2022)^18^ developed a flexible and sustainable analytical device with the ability to satisfy the SDGs from Agenda 2030, employing PLA as support/substrate for plant-wearable sensors.^18^ With the goal of promoting diagnosis, prompt decision-making, and medical interventions for human applications, the strategy used biodegradable and environmentally friendly gadgets for on-site, selective, rapid, and decentralized monitoring of individuals health status using portable systems.^33^ Biodegradable bio-based PLA films derived from renewable resources were created using the casting method to provide flexible and sustainable substrates/supports. The substrates were used in portable devices and sensing applications as a sustainable alternative for traditional polymers derived from nonrenewable resources. The adaptable and flexible strip sensor with a full electrochemical sensing system built on PLA sustainable substrates by screen printing technology allowed fast, decentralized molecular biomarker testing in non-invasive samples.

## Cellulose Acetate

CA is a sustainable and biodegradable modified natural polymer made of renewable resources such as wood pulp, cotton fibers, or other plant-based sources.^32^ CA is a versatile and cost-effective material with noteworthy properties, such as processability, mechanical strength, hydrophobicity, transparency, and biocompatibility, exhibiting potential applications ranging from packaging to biomedical devices.^58–61^ Furthermore, CA’s natural capacity to biodegrade under the ideal conditions increases its allure as a green alternative due to its environmental friendliness and ability to adapt to individual requirements. The authors of the current perspective have a great deal of experience creating substrates using CA as a sustainable substance covering features, methods of production, and possible benefits until applications, especially in plant-wearable sensor.^18,19,32,62–64^ The biodegradable plant-wearable sensor for pesticide monitoring is a breakthrough in food safety and precision agriculture.^18,19^ The combination of printed electronics and environmentally friendly biopolymeric films resulted in plant-wearable sensors allowing on-site, rapid, and decentralized analysis of pesticides.^19^ The study showed how to create flexible and environmentally friendly sensors printed on CA substrates to detect carbendazim and paraquat in food, water, and agricultural samples. Plant-wearable sensors were manufactured by depositing the entire electrochemical system using the screen-printing technique (SPE) on top of the biodegradable CA substrates prepared by the casting method.^19^ Square wave voltammetry (SWV) and differential pulse voltammetry (DPV) were used to assess the analytical performance, with robust results without interference from other pesticides. The flexible and sustainable non-enzymatic plant-wearable sensor was able to detect the residues of carbendazim and paraquat directly on the skins of lettuce and tomatoes, as well as in water samples. The sensors had a consistent fast response, and were strong and stable in the face of numerous flexions. The plant-wearable sensor offers prospective applications in biomarker detection in human biofluids and on-site hazardous chemical substance analysis due to its high sensitivity, selectivity, ease of use, and fast agrochemical detection.

The use of CA as a substrate for controlled delivery of essential micronutrients in soil and plants was investigated by Callaghan et al.^65^ in which the authors produced zinc-impregnated CA beads using a unique approach with the potential to be scaled up.^65^ Antisolvents containing zinc were used to stimulate absorption during regeneration, which resulted in higher concentrations of the chemicals encapsulated in zinc. The investigation tested several variables, such as the use of zinc salts whose counter-anions had lower radial charge densities, varying CA concentrations, and impregnating CA solutions with zinc acetate. The zinc salts formed had a significant impact on the amount of zinc in the beads and release time in aqueous conditions. Conductivity measurements were used to track zinc release. Surprisingly, the zinc amount impregnation attained was the highest of the literature, and the beads’ release periods were longer than those of current zinc delivery techniques. Zinc sulfate and zinc acetate beads showed sluggish delivery over time in release tests carried out in soil, suggesting the possibility of delivering zinc ions to the soil for periods longer than 45 days. These results showed that a biodegradable carrier can be used to produce a controlled release of micronutrients, potentially eliminating the requirement for polymers derived from petrochemical sources commonly used in agriculture.

## Poly(hydroxy alkanoate)s

A class of biodegradable polymers known for varied chemical properties and extensive applications is polyhydroxyalkanoates or PHAs. Due to their biocompatibility and biodegradability, the PHA polymers—which are produced by microbes as intracellular storage compounds—offer environmentally beneficial substitutes for traditional plastics.^66^ PHAs are suited for a wide range of applications, such as packaging, agricultural products, and biomedical devices, due to their broad spectrum of properties, including flexibility, thermal stability, and chemical resistance.^67^

Polyhydroxybutyrate (PHB) is one of the well-known PHAs used for a variety of applications due to its thermoplastic, transparent, and similar properties to traditional plastics.^32,68^ PHAs also have potential applications in wearable sensors for plant monitoring due to environmental resilience and plant ecosystem compatibility. Other PHA variations with special features to meet certain monitoring demands, such as polyhydroxyvalerate (PHV) and polyhydroxyhexanoate (PHH), also have potential as plant wearable sensors.^33,69^ Researchers hope to create sustainable sensor systems that can improve precision agriculture and encourage environmental stewardship by utilizing the special chemical properties of PHAs.

Rebocho et al. (2020)^70^ used PHA, a byproduct of the fruit processing industry, to create films from apple pulp waste. Co-culturing techniques were employed to enhance substrate consumption efficiency compared to that of monocultures. The resulting PHA mix was processed into flexible and elastic films, which were composed of medium-chain-length PHA (mcl-PHA) and poly­(3-hydroxybutyrate) (P­(3HB)) in roughly equal proportions. The films showed permeabilities to carbon dioxide and oxygen comparable to P­(3HB) films and mechanical properties similar to the mcl-PHA films. This substrate has promising features, including hydrophobicity, for plant-wearable sensor applications.

Mirpoor et al. (2023)^71^ produced substrates using PHA sourced from renewable materials to create innovative bioplastics functionalized with varying concentrations of phloretin, a compound found in fruits and vegetables, to enhance the antioxidant and antimicrobial properties. Lower concentrations of phloretin led to decreased moisture content and swelling ratio values in the films compared to the control due to the hydrophobic nature of phloretin, limiting water retention. Despite variations in phloretin concentration, all films exhibited a low moisture content (less than 6%) and swelling ratio (less than 4%). Moreover, the phloretin addition resulted in increased film hydrophobicity, evidenced by higher contact angle values. The change in wettability is attributed to the hydrophobic properties of phloretin. Film opacity also increased with higher phloretin content, which is an important factor affecting food quality and consumer preferences. Although the primary focus of the study was on the use of these films in packaging, their properties indicate the potential for developing substrates in plant-wearable sensor applications.

## Polybutylene Succinate

PBS is a biodegradable PE produced by the condensation polymerization of succinic acid with 1,4-butanediol. PBS depicts good mechanical properties, including a high modulus and tensile strength, and is highly resistant to heat and chemicals.^72^ PBS is also biocompatible and easily manipulated into a variety of shapes and sizes including films, fibers, and molded items appropriate for a broad range of applications.^73^ Due to the biodegradability, versatility, and sustainability features, PBS has attracted interest as an eco-friendly alternative for biomedical, agriculture, and packaging applications.^24^

PBS-based thin films modified with varied levels of coconut oil (CO) and extra virgin olive oil (EVO) were evaluated to increase the efficacy of food preservation and the polymer properties for food packaging applications.^74^ The mechanical and morphological properties of the films as well as the interactions between the polymer and oils were analyzed by SEM imaging, mechanical testing, and ATR/FTIR spectroscopy. Furthermore, food-contact experiments using wrapped apple and kiwi slices showed that the films may prevent fruit browning and postpone the growth of mold, especially when 3 wt % EVO was added. The ability of these PBS-based films to prolong the shelf life of fresh food and their practical manufacturing demonstrate their potential as environmentally acceptable substitutes for traditional plastic packaging materials.^74^ With advantageous features, PBS-based polymeric materials have great potential to be used as substrates/support for sensor applications in plant monitoring.

## Poly(glycolic acid)

PGA is a biodegradable, renewable, and eco-friendly polymer from the PE family, produced by the polymerization of glycolic acid units, which is a natural molecule found in fruits such as grapes and sugarcane.^31^ PGA has several desirable properties, including mechanical strength and stability properties assigned to the high degree of crystallinity.^75^ Given the high level of biocompatibility, PGA is frequently utilized in biomedical applications such as drug delivery systems, tissue engineering scaffolds, and sutures.^24^

PGA is appropriate for implantable medical devices due to the capacity to gradually break down into non-toxic metabolites inside the body and be easily absorbed by the surrounding tissues without posing a threat.^25^ PGA can be processed using the methods of 3D printing, electrospinning, and melt extrusion, enabling the production of films, scaffolds, and other intricate structures.^76^ Kim et al. (2019)^77^ produced scaffolds using PGA to verify their efficacy in encouraging bone tissue regeneration and investigating possible therapeutic uses for bone defect repair and regeneration. Because of its high mechanical strength and biodegradability, PGA is a recommended material in tissue engineering and is used for scaffold preparation. The effective creation and functionality of PGA scaffolds in promoting bone tissue regeneration point to their versatility for possible integration into wearable sensors. Films appropriate for plant monitoring applications can be produced using the 3D printing process with desired flexibility, durability, and biocompatibility properties essential for wearable sensors.

## Integration of Plant-Wearable Devices with Advanced Technologies

The integration of plant-wearable sensors with advanced technologies such as wireless data transmission, centralized cloud servers, AI, ML, Big Data, and the IoT has the potential to enhance agricultural productivity and minimize economic losses.^20^ Studies have demonstrated the use of innovative technologies combined with sensing devices to monitor crucial parameters for crop growth, including moisture, humidity, temperature, and soil composition.^19,21,33^ Data transmission, whether wired or wireless, is essential for plant-wearable sensors to transfer collected data to devices or cloud servers for repository, visualization, and analysis.^8^ While wired connections simplify the transmission process, they are not ideal for wearable technologies.^8^ Wireless communication technologies, on the other hand, allow for remote monitoring and provide the benefit of transmitting data to mobile gadgets for real-time analysis, enabling on-site decision-making.^8^ Wearable sensors, IoT, and AI collectively possess significant capabilities to acquire and interpret real-time, on-site data for monitoring plant health and crop production.^20^ The integration of sophisticated technologies (IoT, Big Data, and AI) with plant-wearable sensors is transforming traditional agricultural practices into smart farming, featured by enhanced yields.^20^ By translating plant physiological signals into wireless and electrical outputs connected to modern innovative technologies, wearable sensors improve plant health and productivity, thus driving the development of precision agriculture.^20^

## Summary and Outlook

Plant-wearable devices, with their intensifying agriculture precision and food safety importance, show extensive potential for diagnosis, health status, and sustainable practices in plant monitoring applications. Wearable devices will completely revolutionize the agrifood sector, enhancing productivity, decreasing environmental impact, and hunger may fulfill the requirements of the Sustainable Development Goals (SDGs 2, 3, 6, 12 through 15) contained in the United Nations 2030 Agenda. In this perspective, we summarize that the incorporation of sustainable and biodegradable biopolymer-based substrates/supports in the preparation of wearable devices represents a promising approach to developing green, flexible, and transparent plant-wearable sensors. The sustainable wearable devices introduced here for plant monitoring make a valuable and promising alternative to conventional bioelectronics from nonrenewable materials.

Research of biodegradable materials in plant wearable sensor technologies offers a diverse environment with great prospects. Biodegradable sensors are at the forefront of innovation as demand for sustainable alternatives in sensing technology rises in tandem with society’s growing emphasis on environmentally sensitive activities. In the near future, noninvasive and minimally invasive plant-wearable sensors integrated with IoT, AI, ML, and Big Data technologies will revolutionize agriculture precision and food safety fields. We are completely convinced that entire scientific areas are moving to sustainable practices, and further efforts will address most of issues to achieve low cost, flexible, high accuracy, robust, convenience, comfortable, stable, sustainable, and biodegradable wearable devices. Specifically, research on plant-wearable gadgets will also enable new heights.

The main critical challenges in the development of wearable sensors for plant monitoring and precision agriculture are working and storage stability as well as timely analyte detection under varying environmental conditions. An interesting strategy to improve sensor stability is through material selection, including robust polymeric substrates, nanocomposite coatings, or encapsulation techniques, to protect the sensor from environmental impacts, i.e., humidity, UV radiation, and temperature fluctuations. Functionalization surface strategies or modification can enhance sensor longevity and stability by preventing (bio)­fouling or degradation of the active sensing layer. The lifetime storage of sensors is extended by controlling storage conditions, such as humidity and temperature, or with protective layers minimizing degradation before use. The employ of self-healing materials or reversible binding mechanisms is an excellent candidate to maintain sensor integrity over time. Sensor calibration and signal processing techniques, including ML algorithms, can help enhance detection accuracy and compensate for environmental variations. A sensor array with units to measure the humidity and temperature also can be an excellent alternative. Real-time data acquisition systems integrated with wireless transmission ensure prompt detection and analysis, providing insights into plant health.

Future wearable sensor approaches will require high sensitivity and increased storage stability to on-site detection directly on the selected samples under variable environmental conditions.^78^ Plant health status is easily monitored without any damage using wearable sensing technology detecting the key signaling target molecules listed in Table 1.^5,78^ With wearable sensing devices, we can continuously and in real-time track glucose, hydrogen peroxide, calcium ions (Ca^2+^), ethylene, nitric oxide (NO), abscisic acid, jasmonic acid, methyl salicylate, salicylic acid, gallic acid, chlorogenic acid and pesticides imidacloprid, thiram, thiabendazole, parathion-methyl, chlorpyrifos, carbendazim, diquat, diuron, paraquat, and fenitrothion to improve the crop productivity.^5,78^

**Table 1:** Plant Health Status Indicators Monitored with Sensor Technology

target analytes	samples	concentration range	refs
hydrogen peroxide	and *Echeveria Raindrops*	10–100 μM	^15^
glucose	and *Echeveria Raindrops*	50 to 1500 μM	^15^
	L. (sweet pepper), (gerbera), and L. (romaine lettuce)	20 to 80 μM	^12^
sucrose	L.	15–59 μM	^79^
Ca^2+^	different plant species	40 nmol	^80^
NO		0.1–10 μM	^81^
ethylene	kiwifruit	0.1–100 ppm	^82^
jasmonic acid	rice	0.001–0.01 μM	^83^
methyl salicylate		0.01–0.1 ppm	^84^
abscisic acid	tomato leaves	1 nM–100 μM	^85^
pH	and	pH 4–8	^86^
salicylic acid	and	6.6–200 μM	^4^
imidacloprid	honey	0.20–92 μM	^87^
thiram and thiabendazole	leave	10^–4^ to 10^–7^ M	^88^
		10^–5^ to 10^–8^	
parathion-methyl, thiram and chlorpyrifos	apple, orange, and cucumber		^89^
gallic acid and chlorogenic acid	orange and kiwi fruits	0.1–87 μg/mL and 0.1–78 μg/mL	^90^
carbendazim and diquat	cabbage and apple	0–1.4 μM	^18^
carbendazim and paraquat	lettuce and tomato	0.1–1.0 μM	^19^
carbendazim	apple, cabbage, and orange juice	0.1–1.0 μM	^21^
diuron		1–10 μM	
paraquat		1.1–1.0 μM	
fenitrothion		1–10 μM	
